# Key targets and mechanisms by which gut microbiota-derived metabolites regulate Alzheimer’s disease through the immune - inflammatory pathway: Based on network pharmacology and molecular docking

**DOI:** 10.1371/journal.pone.0352999

**Published:** 2026-07-06

**Authors:** Lei Miao, Fengwei Hou, Guanghong Li

**Affiliations:** 1 Heze Medical College, Heze, Shandong, China; 2 Heze Municipal Hospital, Heze, Shandong, China; NCCS: National Centre For Cell Science, INDIA

## Abstract

This study integrated network pharmacology, bioinformatics, and molecular docking to explore potential immune-inflammatory pathways associated with the relationship between gut microbiota-derived metabolites and Alzheimer’s disease (AD). A total of 260 gut microbiota – derived metabolites were initially retrieved, and 196 common targets were identified by intersecting predicted metabolite-associated targets with AD-related targets. Further screening identified 14 key overlapping targets, including IL6, NFKB1, IL1B, PTGS2, TLR4, and PPARG. Protein–protein interaction (PPI) network analysis identified IL6, NFKB1, IL1B, CXCL8, PPARG, FOS, and JUN as central hub genes. Functional enrichment analyses indicated that these targets were mainly involved in immune-inflammatory responses, response to lipopolysaccharide, oxidative stress-related processes, and regulation of apoptosis. KEGG pathway analysis further suggested that the overlapping targets were associated with several inflammation-related signaling pathways, including the NOD-like receptor, TNF, NF-κB, and MAPK signaling pathways. In silico pharmacokinetic and toxicity evaluation showed that several representative metabolites exhibited heterogeneous but informative drug-likeness and pharmacokinetic/toxicity-related profiles relevant to gut–brain-axis hypothesis generation. Molecular docking was performed as an exploratory structural assessment and suggested that selected metabolites, including Enterodiol, Coumarin, and 3,9-dihydroxy-6H-benzo[c]chromen-6-one, showed top-ranked predicted docking poses in computationally identified surface-accessible pockets of representative hub proteins such as IL6 and NFKB1, with docking scores ranging from – 6.8 to – 8.1 kcal/mol. These docking scores were interpreted only as qualitative descriptors of predicted structural compatibility and were not used to infer quantitative biological activity, target inhibition, or therapeutic efficacy. Overall, this study prioritizes a potential multi-target immune-inflammatory network centered on IL6, NFKB1, and IL1B, providing a hypothesis-generating framework for understanding the possible role of gut microbiota-derived metabolites in AD-related neuroimmune regulation. Further experimental studies are required to validate the predicted metabolite–target associations and clarify their biological relevance.

## 1. Introduction

Alzheimer’s disease (AD) is the most common type of dementia, and its clinical manifestations mainly include memory loss, cognitive impairment and daily living ability decline [1,2]. The pathological features are progressive loss of neurons in the cerebral cortex and hippocampus, deposition of β-amyloid (Aβ) plaques [3], abnormal phosphorylation of tau protein to form neurofibrillary tangles [4], accompanied by activation of glial cells and neuroinflammation [5]. Currently, although symptomatic interventions are available, no curative treatment for AD has been established. Therefore, in-depth exploration of AD – related pathological processes from a systems-level perspective and identification of disease-associated pathways and candidate molecular nodes have become important research directions. Over the past decade, researchers have gradually realized that the pathogenesis of AD is not limited to the classical “amyloid plaque - tau tangle - neuronal death” framework in the brain [6], but involves the synergy of systemic metabolism [1], immunity [7], gut-brain axis and other multi-dimension systems [8]. The gut microbiota, which plays a crucial role in host metabolic homeostasis, immune regulation, and maintenance of nervous system function, is increasingly regarded as an important component that may be associated with AD-related pathological processes [9]. A series of studies have shown that the composition and abundance of intestinal flora in AD patients are significantly changed, which is characterized by a decrease in the diversity of intestinal flora, a decrease in beneficial bacteria, and an increase in harmful bacteria. At the same time, the generation of microbial metabolites and the host metabolic environment also change dynamically. More specifically, gut microbial dysbiosis in AD is not merely a compositional shift, but a functional imbalance characterized by depletion of beneficial short-chain fatty acid-producing bacteria and enrichment of pro-inflammatory or endotoxin-associated taxa. Such dysbiosis may impair intestinal barrier integrity, promote peripheral immune activation, and increase the translocation of microbial components such as lipopolysaccharide into the circulation, thereby amplifying systemic and neuroinflammation [10]. In addition, dysbiosis can reshape the host metabolic milieu by altering the production of key microbiota-derived metabolites, including short-chain fatty acids, bile acid derivatives, trimethylamine-N-oxide, and tryptophan-related metabolites [11]. Therefore, gut microbial dysbiosis may provide an important upstream context for the altered metabolite profiles observed in AD, and supports the rationale for focusing on microbiota-derived metabolites as functional mediators along the gut-brain axis.

The gut microbiota-brain axis refers to the mechanism of bidirectional communication between the gut microbiome and the central nervous system through multiple channels such as nervous, immune, blood circulation, and endocrine [12]. Under this mechanism, gut microbiota not only participate in food digestion and nutrient metabolism [13], but also produce abundant metabolites [14]. Some microbial metabolites may enter host circulation and, in specific contexts, have been reported to influence or cross the blood-brain barrier (BBB); however, direct CNS exposure requires experimental validation [15]. Studies have shown that in AD animal models and humans, the imbalance of gut microbiota structure and the changes of microbial metabolites are related to the decline of cognitive function [16], the pathological load of Aβ/tau in the brain, the increase of neuroinflammation level, the destruction of blood-brain barrier, and abnormal lipid metabolism [17].

Importantly, the biological significance of these metabolites should be understood in the context of gut microbial dysbiosis, because alterations in microbial community structure are a major determinant of changes in metabolite production, transformation, and host exposure. Gut microbiota-derived metabolites are small molecular compounds produced or transformed by intestinal microorganisms from substrates such as dietary fiber, amino acids, bile acid derivatives, and lipids [18]. These metabolites include short-chain fatty acids such as acetate, propionate, and butyrate, secondary bile acids, trimethylamine-N-oxide (TMAO), aromatic amino acid metabolites, sphingolipid metabolites, neurotransmitter precursors, and their derivatives [19,20]. A growing body of evidence suggests that these metabolites may contribute to AD-related pathological processes through their effects on host immune regulation, metabolic pathways, nervous-system function, and BBB-related homeostasis [21]. For example, SCFAs can regulate immune cell status and inflammatory response by activating G‑ protein-coupled receptors (e.g., GPR41, GPR43) or histone deacetylase (HDAC) inhibition [22]. Studies have found that AD patients are often accompanied by decreased levels of SCFAs and increased levels of TMAO [23], and this metabolite change is correlated with cognitive scores, brain atrophy, and Aβ/tau biomarkers. In addition, gut microbiota-derived secondary bile acids are more cytotoxic than primary bile acids and have been shown to be associated with elevated gamma-secretase activity, increased Aβ production, and cognitive decline [24].

It is of great significance to include metabolites of gut microbiota in the study of the pathogenesis of AD. This perspective helps extend AD research from local brain abnormalities to systemic gut-brain-metabolic-immune interactions, thereby supporting a systems-biology understanding of the disease. As detectable and potentially modifiable mediators, gut microbiota-derived metabolites may provide an informative direction for future biomarker discovery and microbiome-related research in AD [25].

In this context, by systematically collecting metabolites, predicting relevant targets, constructing a “metabolite-target-disease” network, and combining protein interaction analysis, functional enrichment analysis, and pharmacokinetic/toxicity assessment. Network pharmacology can be used to prioritize candidate metabolite-target associations and generate mechanistic hypotheses regarding the relationship between microbial metabolites and AD. This method has been applied in metabolic diseases and remains less extensively explored in AD-related gut-brain-axis research. Therefore, this study aimed to prioritize candidate targets, pathways, and representative metabolites associated with the potential relationship between gut microbiota-derived metabolites and AD-related immune-inflammatory regulation. Protein interaction analysis, functional enrichment analysis, and in silico pharmacokinetic/toxicity assessment were integrated to provide a hypothesis-generating framework for future validation studies.

## 2. Materials and Methods

### 2.1. Identification of gut microbiota-derived metabolites and their target genes

Firstly, gut microbiota-derived metabolites and their corresponding gut-related target genes were obtained from the gutMGene v2.0 database. All metabolites were standardized and converted into SMILES format using PubChem and RDKit for subsequent target prediction. Putative targets of the metabolites were predicted using SEA (Similarity Ensemble Approach) and SwissTargetPrediction. Only targets restricted to Homo sapiens were retained. To improve the reliability of prediction, only targets meeting the preset confidence criteria in each platform and supported by both SEA and SwissTargetPrediction were kept for downstream analysis. After removal of duplicates, the overlapping target set was defined as the candidate metabolite-associated target set.

In addition, because target prediction results are computationally inferred and may be influenced by database coverage and algorithmic bias, an intersection strategy was adopted to increase stringency and reduce false-positive predictions.

### 2.2. Identification of Alzheimer’s disease-related targets

Disease-related targets for Alzheimer’s disease were collected from GeneCards, CTD, and DrugBank using “Alzheimer’s disease” as the keyword. To improve disease relevance and reduce noise, only GeneCards targets with a relevance score ≥30 and CTD targets with an inference score ≥50 were retained, while curated AD-related targets recorded in DrugBank were also included. After merging and deduplication, the resulting set was used as the AD-associated target set for subsequent overlap analysis.

### 2.3. Construction and analysis of PPI network

The intersection targets were submitted to the STRING (Search Tool for the Retrieval of Interacting Genes/Proteins) database, and the combined score threshold was set to 0.4. A protein–protein interaction (PPI) network was constructed for the overlapping targets between gut microbiota-derived metabolite-associated targets and AD-associated targets. The interaction between targets was visualized using Cytoscape software. Based on the topological analysis of PPI network, degree centrality was used to identify the most influential hub genes in the network. These genes were considered candidate hub nodes potentially associated with AD-related biological processes.

### 2.4. GO and KEGG enrichment analysis

DAVID (Database for Annotation, Visualization and Integrated Discovery) was used to perform Gene Ontology (GO) and KEGG pathway enrichment analyses of the key overlapping targets. Biological process (GO-BP), cellular component (GO-CC), and molecular function (GO-MF) categories were analyzed. P values were adjusted using the Benjamini-Hochberg method, and terms with adjusted P values or false discovery rate (FDR) < 0.05 were considered statistically significant. A minimum gene count threshold of ≥ 5 was applied in KEGG enrichment analysis. In addition to statistical significance, fold enrichment and gene ratio were also recorded to improve the interpretability of the enrichment results. Unless otherwise specified, the default Homo sapiens background gene universe provided by the enrichment platform was used as the reference set.

### 2.5. Metabolic kinetics and toxicity assessment

The pharmacokinetic properties of gut microbiota-derived metabolites were evaluated using SwissADME and ADMETlab platforms. According to Lipinski’s Rule of Five, molecular weight (MW), hydrogen-bond donors (HBD), hydrogen-bond acceptors (HBA), hydrophobicity-related parameters such as LogP, and other descriptors were assessed to preliminarily characterize in silico drug-likeness and pharmacokinetic properties. Potential toxicity liabilities, including carcinogenicity, hepatotoxicity, and cardiotoxicity-related hERG inhibition, were preliminarily estimated using the ADMETlab platform.

### 2.6. Construction of the microbiota–substrate–metabolite–target (M-S-M-T) network

We constructed a microbiota-substrate-metabolite-target network to summarize putative associations among gut microbiota, substrates, metabolites, and AD-associated targets. Through this network, we summarized candidate relationships between AD-associated hub genes and selected metabolites. Cytoscape (version 3.9.1) was used to visualize the microbiota-substrate-metabolite-target (M-S-M-T) network. The network was constructed as an association-based integrative framework to summarize putative links among gut microbes, substrates, metabolites, and AD-related targets.

## 3. Results

### 3.1. Systematic screening of gut microbiota-derived metabolites and targets related to AD

#### 3.1.1. Identification of candidate targets of gut microbiota-derived metabolites.

A total of 260 gut microbiota-derived metabolites were initially retrieved from gutMGene v2.0 database. After structural integrity verification, 217 metabolites that could be converted into the SMILES format were retained, and 117 gut-related target genes were obtained. In order to comprehensively predict the potential targets of metabolites, SEA and STP databases were used for target prediction, among which 1286 targets were predicted by SEA database and 1281 targets were predicted by STP database. Intersection analysis of the prediction results from the two databases using an online Venn diagram tool identified 838 overlapping targets. These targets were defined as candidate targets of gut microbiota-derived metabolites, which increased the stringency of the downstream analysis.

#### 3.1.2. Collection and screening of disease targets related to AD.

With “Alzheimer’s disease” as the core keyword, the AD related disease targets were systematically searched in three authoritative databases, GeneCards, CTD and DrugBank. To ensure the relevance between targets and diseases, targets with correlation scores ≥30 were screened from GeneCards database. After deduplication and integration, 871 AD-associated targets were obtained. Overlap analysis of 838 candidate targets of gut microbiota-derived metabolites obtained from the above screening with 871 AD-related targets yielded 196 common targets, which provided key clues for the potential association between gut microbiota-derived metabolites and AD.

#### 3.1.3. Identification of key overlapping targets.

**Fig 1:**
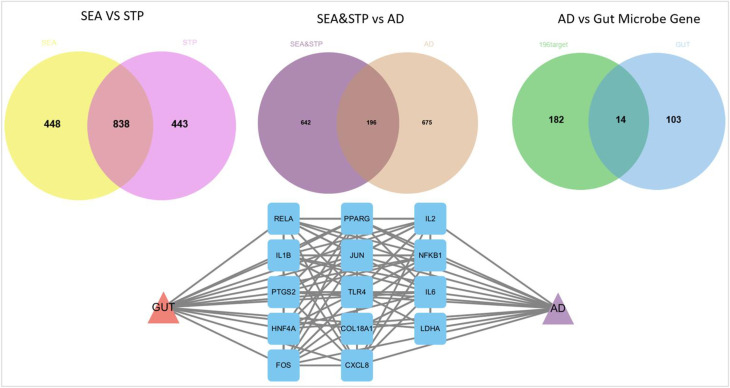
Screening workflow and gut microbiota metabolite-target-Alzheimer’s disease (AD) interaction network. Venn diagrams show target overlaps among SEA, STP, AD-related genes, and gut-related genes. The network highlights 14 key overlapping targets linking gut microbiota-derived metabolites to AD-associated immune-inflammatory regulation.

The 196 common targets were further overlapped with 117 gut related genes, and 14 key overlapping targets were screened out, including RELA, IL1B, PTGS2, HNF4A, FOS, PPARG, JUN, TLR4, COL18A1, CXCL8, IL2, NFKB1, IL6 and LDHA. Based on these key targets, a gut microbiota-derived metabolite-target-AD association network was constructed, as shown in Fig 1, to summarize the putative relationships among metabolites, targets, and AD. These genes occupied central positions in the network and may represent candidate nodes for future experimental validation of gut microbiota-derived metabolite-related mechanisms in AD.

### 3.2. PPI network construction and core hub gene analysis

**Fig 2:**
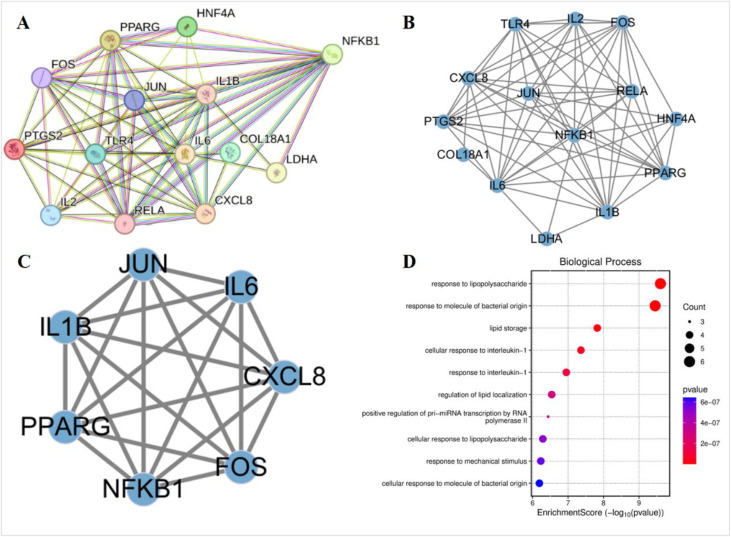
Protein-protein interaction (PPI) network and core module of key overlapping targets. (A) STRING PPI network. (B) Cytoscape visualization and hub-gene screening. (C) Core cluster containing IL6, NFKB1, IL1B, CXCL8, PPARG, FOS, and JUN. (D) Gene Ontology biological process (GO-BP) enrichment of the core cluster, mainly involving lipopolysaccharide response, bacterial-molecule response, inflammatory regulation, and oxidative stress.

The 14 key overlapping targets were submitted to the STRING database with Homo sapiens as the selected species and an interaction score threshold of >0.4. The resulting PPI network was visualized using Cytoscape. The results show that this PPI network contains 14 nodes and 66 edges, and the network density is high, suggesting a close interaction between each target, as shown in Fig 2A. Based on degree centrality (DC), nodes in the network were ranked, and genes with DC values greater than 10 were selected as hub genes, as shown in Fig 2B. These genes occupy key positions in the network and may be the core action nodes of gut microbiota-derived metabolites in the regulation of AD. Functional module mining of the PPI network resulted in a core functional cluster with 7 targets and 21 edges, as shown in Fig 2C. GO biological process enrichment analysis was performed on this cluster, as shown in Fig 2D, it was mainly enriched in the response to lipopolysaccharide, response to bacterial-derived molecules, inflammatory response regulation, oxidative stress response, cell response to biological stimulation, and cell response to interleukin-1, and other pathways. FDR values of enriched terms were all less than 0.05, which was statistically significant. These genes occupied central positions in the network and may represent candidate nodes for future experimental validation of gut microbiota-derived metabolite-related mechanisms in AD.

### 3.3. GO enrichment analysis

**Fig 3:**
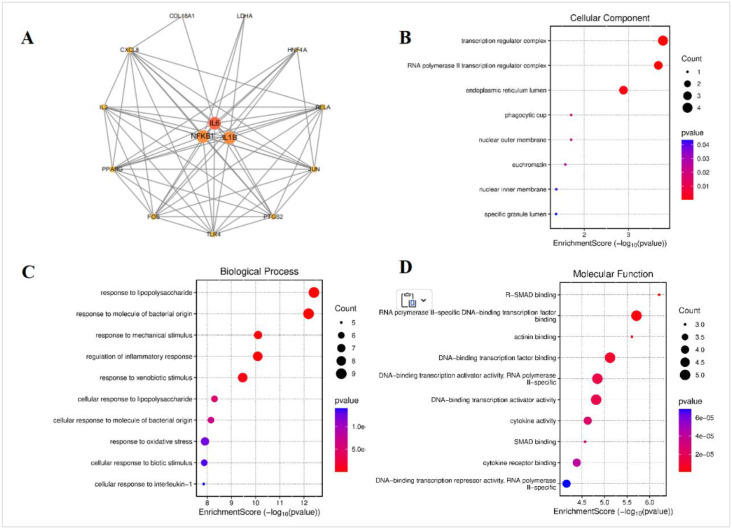
Gene Ontology (GO) enrichment analysis of core Alzheimer’s disease (AD)-related targets regulated by gut microbiota-derived metabolites. (A) Hub-gene network. (B) GO cellular component (GO-CC) enrichment. (C) GO biological process (GO-BP) enrichment. (D) GO molecular function (GO-MF) enrichment. The results indicate enrichment in inflammatory signaling, bacterial-molecule response, transcriptional regulation, and cytokine or receptor binding.

Comprehensive Gene Ontology (GO) enrichment analysis was performed on 14 key overlapping targets to deeply analyze their functional characteristics. As shown in Fig 3, at the biological process (GO-BP) level, in addition to the enrichment pathways related to functional clustering mentioned above, the core targets were also significantly enriched in transcription regulation (such as transcription factor activity regulation, RNA polymerase Ⅱ mediated transcription regulation), apoptosis regulation, oxidative stress response, immune cell activation and other processes. Among these pathways, “regulation of inflammatory response” and “response to lipopolysaccharide” were the most enriched (-log₁₀(FDR) > 10), which further supported the association between immune-inflammatory processes and the predicted gut microbiota-derived metabolite-related target network in AD. At the cellular component (GO-CC) level, the core targets were mainly enriched in cell membrane, cytoplasm, nucleoplasm, inflammasome, transcription factor complex, extracellular matrix and other structures. Among them, the enrichment of “inflammasome” and “cell membrane receptor complex” suggests that the core targets may participate in the initiation and amplification of inflammatory response through cell membrane receptor-mediated signal transduction or the formation of inflammasomes, thereby potentially contributing to AD-related immune-inflammatory processes. At the level of molecular function (GO-MF), the core targets were mainly involved in cytokine binding, receptor binding, protein homodimer binding, transcription factor binding, and enzyme activity regulation. For example, targets such as IL6 and IL1B have cytokine activity and can activate downstream signaling pathways by binding to their corresponding receptors. As transcription factors, PPARG, JUN and other targets can regulate the expression of downstream target genes and participate in the process of metabolism and inflammation. The enrichment of these molecular functions provides supportive information for developing hypotheses about how gut microbiota-derived metabolites may be associated with AD-related immune-inflammatory regulation.

### 3.4. KEGG enrichment analysis

**Fig 4:**
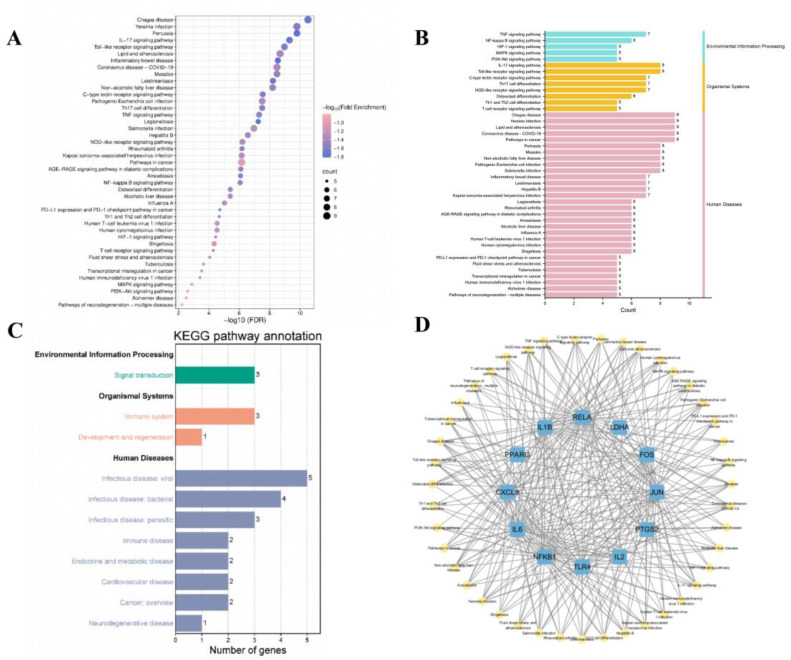
KEGG enrichment analysis of core Alzheimer’s disease (AD)-related targets regulated by gut microbiota-derived metabolites. (A) Dot plot of significantly enriched KEGG pathways based on the key overlapping targets. The y-axis shows the pathway names, the x-axis shows −log10(FDR), bubble size indicates the gene count, and bubble color indicates the enrichment metric shown in the color scale. (B) Functional classification of the significantly enriched KEGG pathways. Pathways are grouped into major KEGG categories, and bar length represents the number of genes enriched in each pathway. (C) KEGG pathway annotation analysis showing the distribution of enriched pathways across different annotation classes. The x-axis represents the number of genes assigned to each category. (D) Target–pathway interaction network of representative core genes and enriched KEGG pathways. Blue square nodes denote target genes, yellow circular nodes denote pathways, and connecting lines denote the associations between targets and pathways. The network illustrates that multiple hub genes participate in several interconnected immune-inflammatory and AD-related pathways.

The key signaling pathways involved in the core targets were revealed. KEGG pathway enrichment analysis was performed on 14 key overlapping targets, and more than 43 significantly enriched pathways (adjusted p-value < 0.05, number of genes > 5) were obtained by enrichment analysis, as shown in Fig 4. The core pathways include NOD-like receptor signaling pathway, TNF signaling pathway, inflammatory bowel disease related pathway, phosphatidylinositol signaling pathway, MAPK signaling pathway, NF-κB signaling pathway, etc. These pathways are closely related to the regulation of immune inflammation, cell apoptosis and metabolic disorders, and play an important role in the pathological process of AD. Notably, in the context of AD-associated neuroinflammation, these enriched pathways are generally interpreted as being predominantly overactivated rather than suppressed [26]. This interpretation is supported by the identification of core pro-inflammatory targets such as IL6, IL1B, NFKB1, TLR4, and PTGS2, as well as by the enrichment of biological processes related to inflammatory response, response to lipopolysaccharide, and immune activation [27]. Therefore, the KEGG results suggest that gut microbiota-derived metabolites may influence AD progression mainly by modulating aberrantly activated inflammatory signaling cascades. For example, NOD-like receptor signaling pathway can promote the release of pro-inflammatory factors by activating inflammasomes, and TNF signaling pathway can mediate inflammatory response and apoptosis. Abnormal activation of these pathways may aggravate nerve damage in AD. Further KEGG classification and annotation analysis divided the enriched pathways into three functional categories: human diseases (including AD, inflammatory bowel disease, atherosclerosis, etc.), organism systems (immune system, endocrine system), signal transduction (NOD-like receptor signaling pathway, TNF signaling pathway, MAPK signaling pathway, etc.). Among these categories, signal transduction pathways showed prominent enrichment. AD-related human disease pathways were mainly associated with immune-inflammatory or metabolic processes, suggesting that the predicted metabolite-associated targets may be involved in pathways relevant to AD pathogenesis. Based on the above results, we constructed the target-pathway interaction network, and the results showed that the core hub genes such as JUN, IL6, NFKB1, and TLR4 were involved in the regulation of multiple key pathways. For example, IL6 is involved in the regulation of TNF signaling pathway, MAPK signaling pathway and NF-κB signaling pathway at the same time, and JUN is involved in the regulation of MAPK signaling pathway and transcription regulation related pathways. This feature of multi-pathway cross-regulation suggests that the core targets may affect the pathological process of AD by forming a complex signaling network.

### 3.5. Pharmacological properties of key metabolites

To preliminarily characterize selected gut microbiota-derived metabolites, SwissADME and ADMETlab were used to evaluate the in silico pharmacokinetic and toxicity-related properties of 10 representative metabolites, including Enterodiol, Linoleic acid, 10-oxo-11-octadecenoic acid, Taurolithocholic acid, Coumarin, 3,9-dihydroxy-6H-benzo[c]chromen-6-one, Quinic acid, Sedoheptulose, 4-[(Z)-15,16-dihydroxydotriacont-19-enyl]-2-methyl-2H-furan-5-one, and Cohibin B, as shown in Table 1. In addition, because Alzheimer’s disease is a central nervous system disorder, the potential ability of the selected metabolites to cross the blood-brain barrier (BBB) should also be considered. In the present study, BBB permeability was evaluated at the in-silico level as part of the pharmacokinetic assessment. Such prediction may help distinguish metabolites with possible BBB-related properties from those more likely to exert indirect effects through peripheral immune-inflammatory regulation and gut-brain-axis signaling. However, these results should be interpreted as computational estimates rather than direct experimental evidence of BBB penetration. Evaluation of pharmacological properties showed that three of the metabolites met Lipinski’s five rules (molecular weight < 500 Da, hydrogen bond acceptor < 10, hydrogen bond donor ≤5, MlogP ≤ 4.15, and number of rule violations ≤1), as shown in Table 1. All metabolites had bioavailability scores > 0.1 (range 0.55 to 0.85) and topological polar surface area (TPSA) < 140 (range 37.3 to 107.22), suggesting potentially favorable in silico absorption- and bioavailability-related properties.

**Table 1:** In silico drug-likeness and pharmacokinetic descriptors of key metabolites.

Metabolite	MW	HBA	HBD	MLOGP	Lipinski violations	Bioavailability score	TPSA
Enterodiol	302.37	4	4	2.1	0	0.55	80.92
Linoleic Acid	280.45	1	1	5.88	1	0.17	37.3
10-Oxo-11-octadecenoic acid	296.45	2	1	5.29	1	0.17	54.37
Taurolithocholic acid	483.72	4	3	4.43	0	0.55	103.7
Coumarin	146.14	2	0	1.79	0	0.55	30.21
3,9-dihydroxy-6H-benzo[c]chromen-6-one	228.2	4	2	2.36	0	0.55	70.67
Quinic acid	192.17	5	5	−2.32	0	0.55	118.22
Sedoheptulose	210.18	7	6	−4.02	1	0.17	138.45
4-[(Z)-15,16-dihydroxydotriacont-19-enyl]-2-methyl-2H-uran-5-one	576.95	4	2	10.69	2	0.17	66.76
Cohibin B	548.89	4	2	9.91	2	0.17	66.76

To improve the clarity and interpretability of the metabolite analysis, the major gut microbiota – derived metabolites identified in this study were further summarized in Table 2. The table lists metabolite names, chemical classes, possible microbial or substrate-related origins, predicted or associated targets, and potential relevance to AD-related immune-inflammatory regulation. Because the present study was based on database mining and network pharmacology rather than direct metabolomic profiling of control and AD samples, quantitative fold-change values for individual metabolites under control versus AD conditions were not available in the current analytical framework.

**Table 2:** Summary of key gut microbiota-derived metabolites, their potential microbial contributors, and AD-related relevance.

Metabolite	Metabolite class	Possible microbial/ substrate origin	Representative microbial taxa potentially involved	Related target(s)	Potential relevance to AD
Enterodiol [28]	Lignan-derived metabolite	Microbial conversion of dietary lignans	*Eubacterium* spp., lignan-transforming gut bacteria	IL6, NFKB1	Modulation of immune-inflammatory signaling; potential neuroprotective effect
Linoleic acid [29]	Polyunsaturated fatty acid	Host–microbial co-metabolism of dietary lipids	Lipid-metabolizing gut microbes, *Collinsella aerofaciens*	PPARG, IL6	Involved in lipid metabolism and inflammatory regulation
10-oxo-11-octadecenoic acid [29]	Oxidized fatty acid metabolite	Microbial oxidation of unsaturated fatty acids	Lipid-metabolizing gut microbes	PPARG, IL6	Associated with oxidative stress and inflammation
Taurolithocholic acid [30]	Secondary bile acid derivative	Microbial transformation of primary bile acids	*[Clostridium] scindens*, *Eubacterium* spp.	IL1B, NFKB1	Linked to bile acid dysregulation and neuroinflammation
Coumarin [31]	Phenolic compound	Microbial biotransformation of plant-derived substrates	Polyphenol-transforming gut microbes	NFKB1	Potential anti-inflammatory activity via NF-κB pathway
3,9-dihydroxy-6H-benzo[c]chromen-6-one [31]	Polyphenol-derived metabolite	Microbial metabolism of aromatic compounds	Polyphenol-metabolizing gut microbes	NFKB1	May regulate inflammatory signaling pathways
Quinic acid [32]	Organic acid/ polyphenol-related metabolite	Microbial metabolism of plant-derived polyphenols	Polyphenol-transforming gut microbes	IL6, IL1B	Associated with metabolic regulation and inflammation
Sedoheptulose [32]	Carbohydrate-related metabolite	Microbial carbohydrate metabolism and pentose phosphate pathway	Carbohydrate-fermenting gut microbes	IL6	Involved in energy metabolism and cellular stress response
4-[(Z)-15,16-dihydroxydotriacont-19-enyl]-2-methyl-2H-uran-5-one	Lipid-related metabolite	Putative microbial lipid transformation	Long-chain lipid-transforming gut microbes	Not specified	Candidate metabolite with potential metabolic regulatory role
Cohibin B	Complex lipid-like metabolite	Putative microbial biotransformation of complex lipids	Complex lipid-metabolizing gut microbes	Not specified	Candidate metabolite; functional role requires further validation

### 3.6. Association-based microbiota-substrate-metabolite-target (M-S-M-T) network analysis

**Fig 5:**
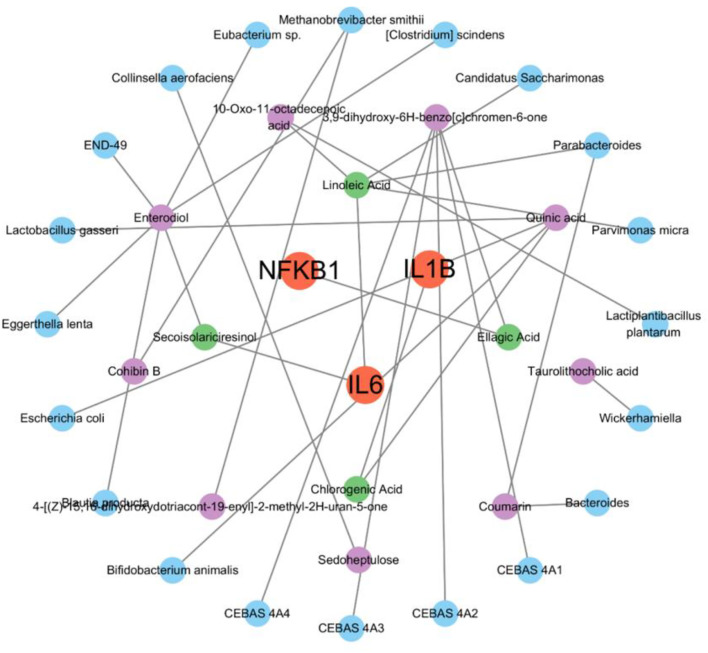
Association-based microbiota-substrate-metabolite-target (M-S-M-T) network. The network links representative gut microbes, substrates, metabolites, and inflammatory targets including IL6, IL1B, and NFKB1, illustrating a putative route from microbial metabolism to Alzheimer’s disease (AD)-related immune-inflammatory regulation.

To systematically summarize the potential relationships among gut microbiota, metabolic substrates, metabolites, and host targets, we constructed an association-based microbiota – substrate – metabolite – target (M-S-M-T) network. This network was intended to provide an integrative conceptual framework linking microbial metabolism and host inflammatory targets relevant to AD, rather than a quantitative mechanistic model. The network integrates four key components: 22 gut microbes, including Methanobrevibacter smithii, Eubacterium sp., [Clostridium] scindens, and Collinsella aerofaciens; 10 major metabolic substrates, including quinic acid, coumarin, sedoheptulose, cholesterol, and chlorogenic acid precursors; several selected metabolites; and three core targets, including IL6, IL1B, and NFKB1, as shown in Fig 5. Besides, it should also be noted that microbiota-derived metabolites do not act independently of the microbial ecological context. Their abundance and biological effects are closely linked to gut microbial dysbiosis, which is increasingly recognized as an upstream event in AD. In this regard, our metabolite-centered strategy should be interpreted as a functional downstream extension of dysbiosis research rather than a replacement for direct microbial compositional analysis [33]. Future studies combining microbiome profiling with metabolomics will help clarify how specific dysbiotic patterns are associated with metabolite alterations and immune-inflammatory responses in AD.

### 3.7. Molecular docking of key metabolites to core targets

To further explore the potential structural compatibility between representative gut microbiota-derived metabolites and immune-inflammatory hub proteins, molecular docking analysis was performed for selected metabolite–target pairs. IL6 and NFKB1 were selected as representative targets because they were identified as central nodes in the PPI network and were involved in multiple inflammation-related KEGG pathways. It should be noted that these proteins were not selected as classical small-molecule drug targets. Rather, docking analysis was used as an exploratory computational approach to provide structural clues for prioritizing potential metabolite–target associations.

Based on the preceding pharmacokinetic evaluation and metabolite–target network analysis, Enterodiol, Coumarin, and 3,9-dihydroxy-6H-benzo[c]chromen-6-one were selected for docking with IL6 or NFKB1. The crystal structures of IL6 and NFKB1 were obtained from the RCSB PDB database, using 1ALU for IL6 and 1SVC for NFKB1. The three-dimensional structures of the metabolites were generated from their SMILES formats and subjected to energy minimization before docking. Molecular docking was performed using CB-Dock2 under the default blind-docking settings. The predicted cavities were regarded as computationally identified surface-accessible pockets rather than experimentally validated active or regulatory sites. The top-ranked docking pose for each metabolite–target pair was selected for visualization and qualitative interpretation.

**Fig 6:**
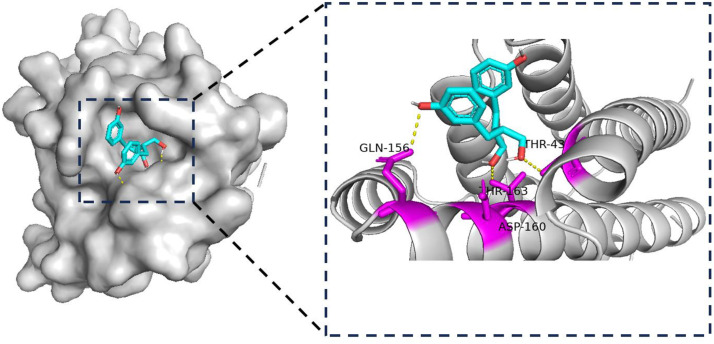
Molecular docking of Enterodiol with IL6 (interleukin-6; PDB ID: 1ALU). The predicted docking pose shows Enterodiol located in a surface-accessible pocket of IL6, with a docking score of −7.2 kcal/mol. This result indicates predicted structural compatibility only.

**Fig 7:**
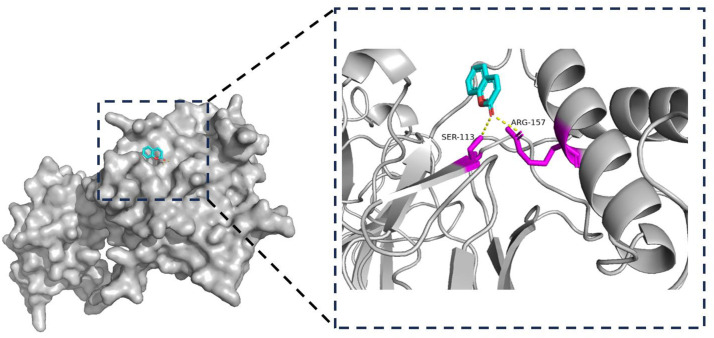
Molecular docking of Coumarin with NFKB1 (nuclear factor kappa B subunit 1; PDB ID: 1SVC). Coumarin fits into a predicted NFKB1 surface pocket with a docking score of −6.8 kcal/mol, suggesting exploratory structural compatibility.

**Fig 8:**
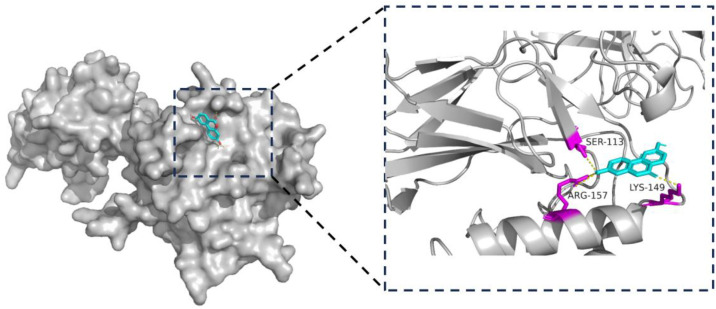
Molecular docking of 3,9-dihydroxy-6H-benzo[c]chromen-6-one with NFKB1 (nuclear factor kappa B subunit 1; PDB ID: 1SVC). The ligand shows a predicted docking pose in the NFKB1 surface pocket with a docking score of −8.1 kcal/mol, indicating predicted structural compatibility only.

The docking results are summarized in Table 3 and shown in Fig 6, Fig 7 and Fig 8. Enterodiol was predicted to adopt a docking pose in a computationally identified surface pocket of IL6, with a docking score of −7.2 kcal/mol. This result suggests that Enterodiol may exhibit spatial compatibility with a computationally predicted pocket of IL6. However, because IL6 is a soluble cytokine that mainly functions through receptor complex formation with IL6R and gp130, this docking result should not be interpreted as evidence that Enterodiol directly blocks IL6–IL6R/gp130 signaling. Coumarin was predicted to adopt a docking pose in a computationally identified pocket of NFKB1, with a docking score of −6.8 kcal/mol, whereas 3,9-dihydroxy-6H-benzo[c]chromen-6-one showed a docking score of −8.1 kcal/mol with NFKB1. These docking scores indicate possible structural compatibility between the selected metabolites and predicted surface regions of NFKB1. Nevertheless, because NFKB1 is a transcription factor whose biological activity is mainly regulated by upstream signaling, dimerization, nuclear translocation, and DNA/protein interactions, these docking results do not demonstrate direct modulation of NFKB1 transcriptional activity.

**Table 3:** Molecular docking results of selected gut microbiota-derived metabolites with representative hub proteins.

Target protein	PDB ID	Metabolite	Docking score (kcal/mol)	Interpretation
IL6	1ALU	Enterodiol	−7.2	Predicted structural compatibility with a computationally identified IL6 surface pocket
NFKB1	1SVC	Coumarin	−6.8	Predicted structural compatibility with a computationally identified NFKB1 surface pocket
NFKB1	1SVC	3,9-dihydroxy-6H-benzo[c]chromen-6-one	−8.1	Predicted structural compatibility with a computationally identified NFKB1 surface pocket

Docking scores were used only for qualitative comparison of predicted docking poses. They should not be interpreted as quantitative evidence of biological activity, target inhibition, or therapeutic efficacy.

Importantly, the docking scores reported in this study were used only as qualitative descriptors of predicted binding poses and structural compatibility. They were not used to infer quantitative biological activity, target inhibition, or therapeutic efficacy. Because experimentally measured activity data were not available for these metabolite-target pairs, no statistical correlation analysis between docking score and biological activity was performed. In addition, molecular dynamics simulations were not conducted in the present study; therefore, RMSD distributions and representative dynamic trajectories were not available. Taken together, the docking results should be considered exploratory structural observations that may help prioritize metabolite–target pairs for future validation by biochemical binding assays, mutational analysis, and cell-based functional experiments.

## 4. Discussion

This study used an exploratory integrative strategy combining network pharmacology, bioinformatics, and molecular docking to prioritize candidate immune-inflammatory targets and pathways through which gut microbiota-derived metabolites may be associated with AD. The analysis highlighted a putative immune-inflammatory network centered on hub genes such as IL6, NFKB1, and IL1B and its association with pathways including NOD-like receptor, TNF, and NF-κB signaling. These findings provide hypothesis-generating systems-level observations for investigating gut–brain axis metabolic–immune crosstalk in AD.

IL-6 not only serves as a key mediator in acute responses but also drives the pro-inflammatory phenotype conversion of microglia by activating JAK/STAT and MAPK pathways, thereby promoting a positive feedback loop of neuroinflammation [6,34]. NF-κB, as a central transcription regulator, is activated in response to multiple injury signals, thereby regulating the expression of numerous pro-inflammatory mediators including TNF-α, IL-1β, and COX-2 [35–37]. Its sustained activation is directly associated with synaptic dysfunction and neuronal apoptosis in AD. IL-1β, massively produced by activated microglia and peripheral immune cells, disrupts blood-brain barrier integrity, enhances γ-secretase activity to promote Aβ generation [6], and directly induces neuronal apoptosis. PPI network and module analysis in this study further revealed that these targets do not act in isolation but form a highly interconnected, functionally synergistic core network module. These results suggest that gut microbiota-derived metabolites may be linked to the inflammatory core module through multiple predicted target associations, providing a basis for future validation of their potential role in AD-related neuroimmune regulation [38,39]. KEGG pathway enrichment analysis further localized core targets to several signaling pathways critical in AD neuroinflammation. The NOD-like receptor pathway recognizes microbial and damage-associated molecular patterns, driving caspase-1-dependent IL-1β/IL-18 maturation and release by activating inflammasomes [40,41]. The TNF signaling pathway directly regulates the cell survival/apoptosis balance and activates downstream NF-κB and MAPK cascades [42]. The NF-κB and MAPK pathways themselves serve as central hubs for the convergence and amplification of multiple inflammatory signals [43]. Their abnormal activation is closely associated with core AD pathological features such as Aβ deposition, tau hyperphosphorylation, and excessive microglial activation. Our findings suggest that gut microbiota-derived metabolites may be involved in gut–brain-axis communication and may relate to immune-inflammatory pathways through peripheral or indirect signaling mechanisms [18,44]. This supports a testable hypothesis that peripheral metabolic signals may be linked to central neuroimmune processes. The docking results also provide preliminary structural observations regarding the different predicted interaction patterns among the selected metabolites. In particular, the coumarin-related metabolites showed distinguishable structural features that may help explain their different docking behaviors in the present computational model. Coumarin and 3,9-dihydroxy-6H-benzo[c]chromen-6-one shared an aromatic lactone-containing scaffold, but they differ in hydroxyl substitution and aromatic extension. Coumarin has a relatively compact structure and limited hydrogen-bond donor capacity, whereas 3,9-dihydroxy-6H-benzo[c]chromen-6-one contains additional hydroxyl groups and a larger aromatic system. These structural features may provide more opportunities for polar contacts and π-related interactions within the predicted NFKB1 surface pocket. This qualitative structural interpretation is consistent with the more favorable predicted docking score of 3,9-dihydroxy-6H-benzo[c]chromen-6-one compared with Coumarin in the current docking model. However, this difference should not be interpreted as a quantitative ranking of biological activity, because no experimentally measured activity or inactivity data were available for these metabolite–target pairs. Enterodiol showed a different structure–interaction pattern from the coumarin-related metabolites. As a lignan-derived metabolite, Enterodiol contains multiple hydroxyl groups and a relatively flexible molecular skeleton. These features may allow it to adapt to a shallow surface-accessible pocket of IL6 through possible polar contacts and conformational accommodation. Nevertheless, IL6 is a soluble cytokine that mainly functions through receptor complex formation with IL6R and gp130 rather than through a classical small-molecule catalytic pocket. Therefore, the predicted docking mode of Enterodiol should be interpreted only as a qualitative structural clue and should not be considered evidence of direct inhibition of IL6 signaling. In contrast, the long-chain lipid-like metabolites, such as 4-[(Z)-15,16-dihydroxydotriacont-19-enyl]-2-methyl-2H-uran-5-one and Cohibin B, showed less favorable overall prioritization in the present analysis despite their potential lipophilic interaction capacity. Their relatively high molecular weight, high MLOGP values, and Lipinski rule violations suggest that excessive hydrophobicity and molecular size may limit their drug-likeness and reduce their suitability for further prioritization in gut–brain-axis-focused follow-up studies. This observation indicates that structural features such as hydrogen-bonding capacity, aromatic extension, molecular polarity, conformational flexibility, and lipophilicity should be considered together when interpreting docking results and metabolite prioritization.

Overall, the present analysis provides a cautious SAR-like interpretation rather than a validated SAR model. The predicted binding modes suggest that hydroxyl substitution and aromatic extension may contribute to more favorable predicted interactions in the NFKB1 pocket, whereas excessive lipophilicity and large molecular size may reduce prioritization despite possible hydrophobic contacts. However, because this study did not include a systematic analogue series with experimentally measured activity or inactivity data, these observations cannot establish a formal structure–activity relationship. Future studies should combine structurally related analogues, molecular dynamics simulations, biochemical binding assays, and cell-based functional experiments to determine whether the predicted interaction patterns correspond to experimentally validated SAR trends. These exploratory structural observations may help prioritize metabolite-target hypotheses for future biochemical, cellular, and in vivo validation [6,45].

Although the gut–brain-axis framework suggests potential CNS relevance, the BBB permeability and CNS exposure of the selected metabolites in the present study remain computationally inferred. We did not experimentally measure metabolite concentrations in brain tissue or cerebrospinal fluid, nor did we validate BBB penetration or direct CNS target engagement. Therefore, the predicted BBB/CNS relevance should be interpreted as hypothesis-generating rather than experimentally validated evidence. Future studies using targeted metabolomics, in vitro BBB permeability assays, cerebrospinal fluid or brain tissue measurements, and in vivo validation are required to confirm the CNS relevance of these candidate metabolites.

## 5. Limitations and future perspectives

Several limitations should be acknowledged. First, this study was based mainly on public databases and computational prediction; therefore, the results may be influenced by database incompleteness, annotation bias, and algorithmic uncertainty. Second, the predicted metabolite–target associations were not experimentally validated, and the actual abundance, tissue distribution, blood–brain barrier permeability, and biological effects of the selected gut microbiota-derived metabolites in AD remain unclear. Third, the molecular docking analysis was exploratory and should not be interpreted as evidence of direct target engagement, target inhibition, or therapeutic efficacy. Because experimentally measured activity data for a systematic analogue series were not available, no validated structure–activity relationship model or statistical correlation between docking scores and biological activity could be established. Moreover, molecular dynamics simulations were not performed; therefore, RMSD distributions and representative dynamic trajectories were not generated. Future studies integrating microbiome and metabolomics datasets, molecular dynamics simulations, biochemical binding assays, and cell-based or animal experiments are required to validate the predicted interactions and clarify their biological relevance in AD-related immune-inflammatory regulation.

## 6. Conclusion

This study integrated network pharmacology, bioinformatics, and molecular docking to prioritize candidate targets, pathways, and metabolite–target associations related to immune-inflammatory regulation in AD. The results suggest that IL6, NFKB1, and IL1B, together with inflammation-related pathways such as the NOD-like receptor, TNF, and NF-κB signaling pathways, may represent candidate molecular components involved in AD-related neuroimmune regulation. Furthermore, the study identified several representative microbial metabolites with informative in silico drug-likeness and pharmacokinetic/toxicity profiles, which may be considered candidates for further experimental validation. This work provides a hypothesis-generating systems-biology framework for future studies integrating microbiome profiling, metabolomics to clarify the potential role of gut microbiota-derived metabolites in AD-related immune-inflammatory regulation.

## Supporting information

S1 File The data used for the present analysis and the corresponding targets are provided in the Supplementary Materials. This study used publicly available databases, and the corresponding access links are provided in Supplementary Table 1.
(ZIP)
